# Supplementary material for: Spatially Resolved Effects of Protein Freeze-Thawing in a Small-Scale Model Using Monoclonal Antibodies
Source: Pharmaceutics. 2020 Apr 21;12(4):382. doi: 10.3390/pharmaceutics12040382 (PMC7238022; doi:10.3390/pharmaceutics12040382)
Supplement: Supplementary file 1 [file pharmaceutics-12-00382-s001.pdf]

pharmaceutics

MDPI

Supplementary Materials: Spatially Resolved Effects of Protein Freeze-Thawing in a Small-Scale Model Using Monoclonal Antibodies

Oliver Spadiut, Thomas Gundinger, Birgit Pittermann and Christoph Slouka *

Table 1. Results on pH in spatial resolved sampling. In blue the minima are highlighted (* percentage change in respect to the starting material).

Sample	pH							
	#1 - 120 min	Change *	#2 - 120 min	Change *	#3 - 240 min	Change *	#4 - NO ramp 12min	Change *
	-	[%]	-	[%]	-	[%]	[g/l]	[%]
Start material	6,080	-	6,167	-	6,246	-	6,226	-
Sample 1	6,031	−0,8	6,221	0,9	6,202	−0,7	6,194	−0,5
Sample 2	5,970	−1,8	6,128	−0,6	6,160	−1,4	6,185	−0,7
Sample 3	6,050	−0,5	6,188	0,3	6,162	−1,3	6,140	−1,4
Sample 4	6,024	−0,9	6,176	0,1	6,185	−1,0	6,195	−0,5
Sample 5	5,930	−2,5	6,125	−0,7	6,079	−2,7	6,162	−1,0
Sample 6	6,084	0,1	6,198	0,5	6,200	−0,7	6,203	−0,4

Table S2. Soluble Aggregates measured by SEC. In green the maxima and in blue the minima are highlighted (* percentage change in respect to the starting material).

Sample	Aggregates - HPLC SEC - 210 nm							
	#1 - 120 min	Change *	#2 - 120 min	Change *	#3 - 240 min	Change *	#4 - NO ramp	Change *
	[%] $^{+}$	[%]	[%] $^{+}$	[%]	[%] $^{+}$	[%]	[%] $^{+}$	[%]
Start material	1,73	-	2,01	-	2,27	-	2,42	-
Sample 1	2,22	28,3	2,31	14,9	2,41	6,2	2,51	3,7
Sample 2	2,16	24,9	2,45	21,9	2,93	29,1	2,58	6,6
Sample 3	1,87	8,1	2,41	19,9	2,45	7,9	2,27	-6,2
Sample 4	1,90	9,8	2,28	13,4	1,84	-18,9	2,51	3,7
Sample 5	2,60	50,3	2,60	29,4	2,81	23,8	2,37	-2,1
Sample 6	1,90	9,8	2,24	11,4	2,08	-8,4	2,61	7,9
**Sample**	**Aggregates - HPLC SEC - 280 nm**							
	#1 - 120 min	Change *	#2 - 120 min	Change *	#3 - 240 min	Change *	#4 - NO ramp	Change *
	[%] $^{+}$	[%]	[%] $^{+}$	[%]	[%] $^{+}$	[%]	[%] $^{+}$	[%]
**Start material**	2,01	-	1,98	-	2,08	-	2,23	-
**Sample 1**	1,99	−1,0	2,07	4,5	2,18	4,8	2,30	3,1
**Sample 2**	2,13	6,0	2,13	7,6	2,45	17,8	2,35	5,4
**Sample 3**	2,13	6,0	2,09	5,6	2,22	6,7	2,36	5,8
**Sample 4**	1,99	−1,0	1,99	0,5	2,14	2,9	2,35	5,4
**Sample 5**	2,20	9,5	2,20	11,1	2,37	13,9	2,47	10,8
**Sample 6**	2,05	2,0	2,02	2,0	2,19	5,3	2,25	0,9

Table S3. Eigenvectors for PCA of Figure 5.

Analyzed Quantity	run	PC 1	PC 2
mAB aggr.	#1	0.24488	0.19663
	#2	0.31343	−0.13294
	#3	0.29187	0.04276
	#4	−0.1202	0.75448
titer HPLC	#1	0.30392	0.18645
	#2	0.30456	0.16524
	#3	0.31757	0.05596
	#4	0.25378	−0.48877
cond.	#1	0.31534	0.07367
	#2	0.30796	0.20006
	#3	0.3171	0.08189
	#4	0.31137	-0.12785
